# Structural Characterization of Glycerol Kinase from the Thermophilic Fungus *Chaetomium thermophilum*

**DOI:** 10.3390/ijms21249570

**Published:** 2020-12-16

**Authors:** Piotr Wilk, Katarzyna Kuśka, Elżbieta Wątor, Piotr H. Małecki, Klaudia Woś, Piotr Tokarz, Grzegorz Dubin, Przemysław Grudnik

**Affiliations:** 1 Malopolska Centre of Biotechnology, Jagiellonian University, 7a Gronostajowa St., 30-387 Kraków, Poland; wilk.piotr@uj.edu.pl (P.W.); katarzyna.kuska@doctoral.uj.edu.pl (K.K.); elzbieta.wator@doctoral.uj.edu.pl (E.W.); klaudia.wos@uj.edu.pl (K.W.); piotr.tokarz@mol.biol.ethz.ch (P.T.); grzegorz.dubin@uj.edu.pl (G.D.); 2 International Institute of Molecular and Cell Biology in Warsaw, 4 Ksiecia Trojdena St., 02-109 Warszawa, Poland; pmalecki@iimcb.gov.pl; 3 Department of Biology, Institute of Molecular Biology & Biophysics, Eidgenössische Technische Hochschule Zürich, Otto-Stern-Weg 5, 8093 Zürich, Switzerland

**Keywords:** glycerol, glycerol kinase, glycerol metabolism, crystal structure, glycerol-3-phosphate, ADP-dependent glucokinase, ADPGK

## Abstract

Glycerol is an organic compound that can be utilized as an alternative source of carbon by various organisms. One of the ways to assimilate glycerol by the cell is the phosphorylative catabolic pathway in which its activation is catalyzed by glycerol kinase (GK) and glycerol-3-phosphate (G3P) is formed. To date, several GK crystal structures from bacteria, archaea, and unicellular eukaryotic parasites have been solved. Herein, we present a series of crystal structures of GK from *Chaetomium thermophilum* (CtGK) in apo and glycerol-bound forms. In addition, we show the feasibility of an ADP-dependent glucokinase (ADPGK)-coupled enzymatic assay to measure the CtGK activity. New structures described in our work provide structural insights into the GK catalyzed reaction in the filamentous fungus and set the foundation for understanding the glycerol metabolism in eukaryotes.

## 1. Introduction

Glycerol metabolism provides a central link between sugar and fatty acid catabolism [1]. In yeast, glycerol can serve as an energy source, providing one ATP molecule from one glycerol molecule. First, it is converted into glycerol-3-phosphate (G3P) by glycerol kinase (GK, Gut1p in *Saccharomyces cerevisiae*), then G3P is transported into mitochondria where it is oxidized to dihydroxyacetone phosphate (DHAP) by glycerol-3-phosphate dehydrogenase (GPD2, Gut2p in *S. cerevisiae*) [2]. The glycerol catabolism not only serves as an alternative energy source but is also important in processes related to signaling, respiration, and lipid synthesis [3,4].

**Figure 1:**
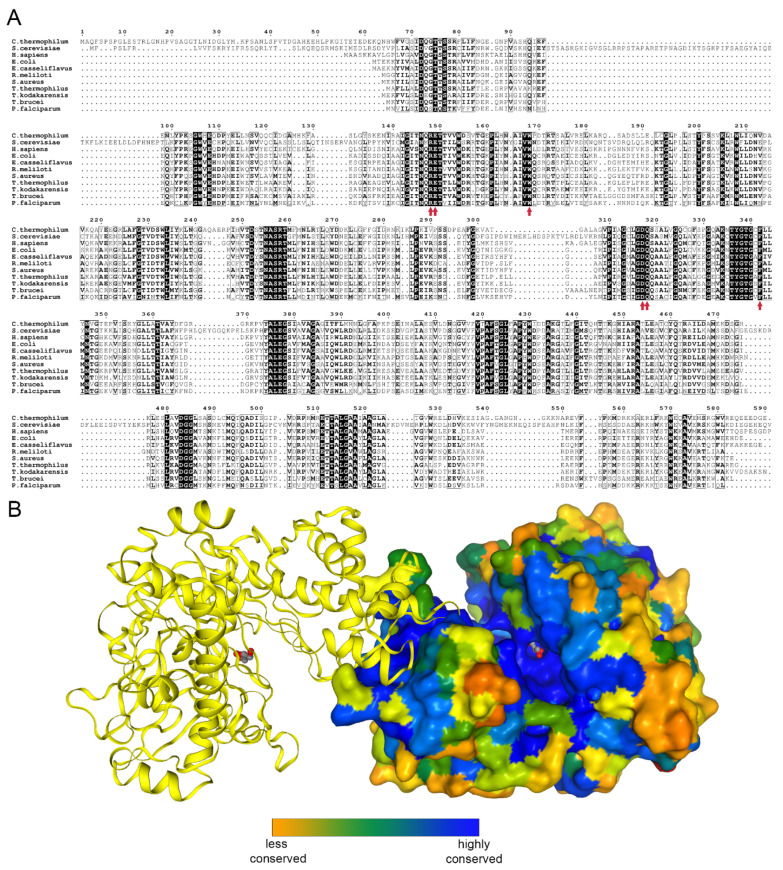
Glycerol kinase conservation. (**A**) Sequence alignment of human, *Saccharomyces cerevisiae*, and all to-date crystalized glycerol kinases prepared using the ENDscript server [6]. The glycerol binding residues are indicated by red arrows. (**B**) A biological dimer of *Chaetomium thermophilum* glycerol kinase (CtGK); one subunit is shown as surface and colored by sequence conservation as analyzed by EPPIC [7].

Glycerol kinase (EC 2.7.1.30) catalyzes the transfer of phosphate moiety from ATP to glycerol to form glycerol 3-phosphate (G3P). The enzyme is conserved among prokaryotes and eukaryotes [5] (Figure 1A,B).

Upon phosphorylation, glycerol cannot pass cell membranes and is therefore retained in the cell. G3P takes part in different metabolic pathways, notably in carbohydrate and lipid metabolism. GK is classified as a member of the sugar kinase family, also known as FGGY kinases, which in turn belongs to the nucleotide binding-sugar kinase/heat shock protein 70/actin superfamily. Proteins belonging to this superfamily are known to undergo large conformational changes upon substrate binding [8,9].

To date, prokaryotic GKs from *Thermus thermophilus* [10], *Escherichia coli* [11,12], *Enterococcus casseliflavus* [13], *Sinorhizobium melilot*, *Cellulomonas* sp., *Elizabethkingia anopheles*, and *Staphylococcus aureus* (unpublished) and the hyperthermophilic archaeon *Thermococcus kodakarensis* [14] are known. These structures confirmed that GK is active as a dimer. In recent years, structures of GK from two unicellular eukaryotic parasites, *Trypanosoma brucei* [15] and *Plasmodium falciparum* [16], were identified as potential antiparasitic targets. The closest available homolog structure to CtGK is one from *Cellulomonas* sp., sharing 46% identity. Herein, we present a series of crystal structures of glycerol kinase from the thermophilic fungus *Chaetomium thermophilum* (CtGK, UniProt ID G0SAG9) in apo form and in complex with glycerol. Furthermore, we used a novel ADP-dependent glucokinase (ADPGK)-coupled assay to measure GK activity.

## 2. Results

### 2.1. Expression, Purification, Stability, and Activity Measurements

**Figure 2:**
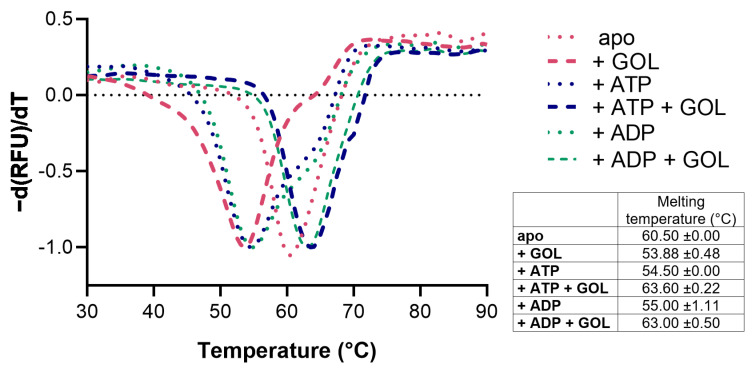
Stability analysis of CtGK presented as a negative first derivative of the normalized fluorescence data. The table summarizes the numerical values of the obtained melting temperatures.

CtGK was expressed using an *Escherichia coli* recombinant system and purified by affinity and size-exclusion chromatography. Next, we assessed CtGK thermal stability by thermal shift assay [17]. The unfolding temperature was determined at 60.50 °C for apoprotein. The addition of glycerol (GOL), ATP, or ADP slightly destabilizes protein and decreases the Tm value to 53.88 ± 0.48 °C, 54.5 ± 0.00 °C, and 55.0 ± 1.11 °C, respectively. While in the simultaneous presence of nucleotide and glycerol, the observed melting temperature increased to 63.6 ± 0.22 °C for the CtGK–ATP–GOL complex and 63.00 ± 0.50 °C for CtGK–ADP–GOL (Figure 2).

**Figure 3:**
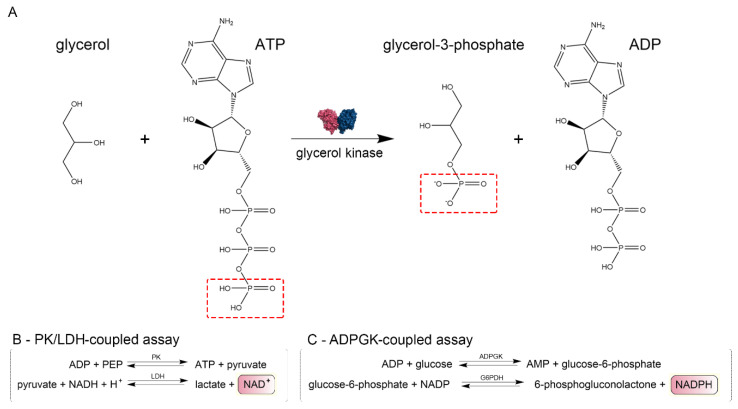
Schematic representation of enzymatic assays. (**A**) Glycerol kinase catalyzed reaction scheme and (**B**,**C**) methods used to assay CtGK activity.

**Figure 4:**
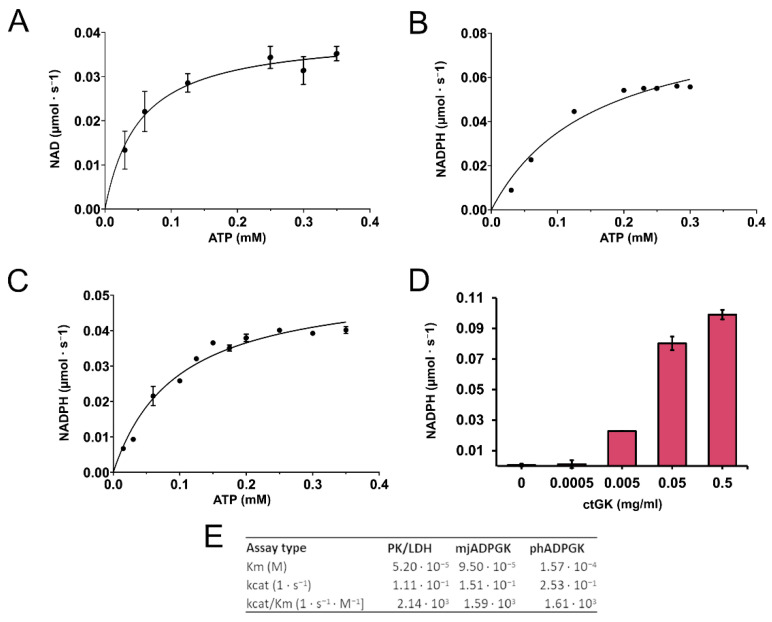
CtGK activity assay. (**A**) Reaction velocity as a function of ATP for CtGK in the PK/LDH-coupled assay, (**B**) phADPGK/G6PD-coupled assay, and (**C**) mjADPGK/G6PD-coupled assay. (**D**) Reaction velocity in the phADPGK/G6PD-coupled assay as a function of the CtGK concentration. (**E**) Kinetic parameters for CtGK-catalyzed reaction measured using different assays.

We confirmed that CtGK exhibits increasing ATPase activity depending on ATP concentration up to around 0.4 mM, and above this value, substrate inhibition was observed. Using a well-established pyruvate kinase/lactate dehydrogenase-coupled activity assay (Figure 3), we measured the apparent Km value for ATP (0.052 ± 0.009 mM) (Figure 4A). Then, encouraged by our previous work on ADP-dependent glucokinases (ADPGKs) [18] and further studies by Ueda and Sakasegawa employing ADPGK to measure creatine kinase activity [19], we decided to test whether ADPGK-coupled enzymatic assay could be used to assess the CtGK activity. ADPGK was first described in hyperthermophilic archaea, where, together with ADP-dependent phosphofructokinase (ADP-PFK), it takes part in a modified glycolysis pathway [20,21]. ADPGK acts in an ADP-dependent manner and replaces hexokinase in phosphorylating glucose to glucose-6-phosphate in the first enzymatic reaction of the glycolytic pathway. In our assay design, ADPGK was used as a sensor of the CtGK catalyzed reaction product, namely ADP, which serves as a substrate for ADPGK. Next, the ADPGK activity was measured in a coupled reaction of glucose-6-phosphate dehydrogenase and the reduction of NADP to NADPH was monitored colorimetrically over the reaction progress (Figure 3C). In our studies, we used the following two ADP-dependent enzymes of archaeal origin: ADPGK from *Pyrococcus horikoshii* (phADPGK) and ADP-dependent gluco/phosphofructokinase from *Methanococcus jannaschii* (mjADPGK/PFK). Using this method, we also confirmed that purified CtGK is active and shows substrate inhibition. The apparent Km values for ATP concentration of 0–0.30 mM were 0.095 ±0.016 and 0.157 ±0.047 for assays using mjADPGK/PFK and phADPGK, respectively (Figure 4B,C).

Previously, it was demonstrated for *E. coli* glycerol kinase that fructose-1,6-bisphosphate (FBP) modulates the activity of GK and favors its tetramerization [11,22]. We analyzed the influence of FBP on properties of CtGK and showed that its presence does not affect the oligomeric state as proven by analytical gel filtration (Figure S1A, Supplementary Materials). However, thermal shift assays have shown that FBP prevents the protein destabilization by GOL and ATP (Figure S1B). This result can suggest the stabilization of one of the CtGK’s domains. Next, we analyzed the effect of FBP on CtGK activity. As it was shown that FBP can allosterically regulate PK [23], as well as it is a known mjADPGK/PFK inhibitor (Figure S2A), we decided to use a phADPGK-coupled assay. Our data show a small inhibitory effect of FBP (retained 87% activity at 5 mM FBP, Figure S2B). However, the inhibitory effect on phADPGK activity is similar, suggesting that the observed decrease may be a result of a subtle inhibition of phADPGK in high FBP concentration (≥5 mM) (Figure S2C).

### 2.2. Overall Structure and Domain Organization

To gain structural insights into mechanisms of glycerol phosphorylation catalyzed by CtGK, we attempted to crystallize it and to solve its structure. CtGK crystallized readily within a few days. Initial molecular replacement efforts using a conventional approach were unsuccessful, we, therefore, decided to solve the structure using a MoRDa automated structure refined pipeline [24]. The first structure was solved in the P3_2_21 space group using a model derived from 5AZI [15]. The asymmetric unit (ASU) contained two polypeptide chains. The electron density map allowed for building the residue ranges 65 (or 67 for chain B) to 583. A short loop of 8 (or 9) amino acids (542–548/9) connecting the α-helix H18 and a β-strand S18 could not be modeled due to insufficient electron density. This likely reflects the high flexibility of this loop. The physiological dimer is not contained in the ASU but completed by a symmetry-related chain forming a rather elongated assembly (A:B’ and B:A’) (Figure 1B). A similar organization was observed for the later structure obtained in P222_1_ (both chains modeled for the range 65–583 with a gap 541–549). The dimer interaction is mostly mediated by β-strands located on the C-terminal domains.

**Figure 5:**
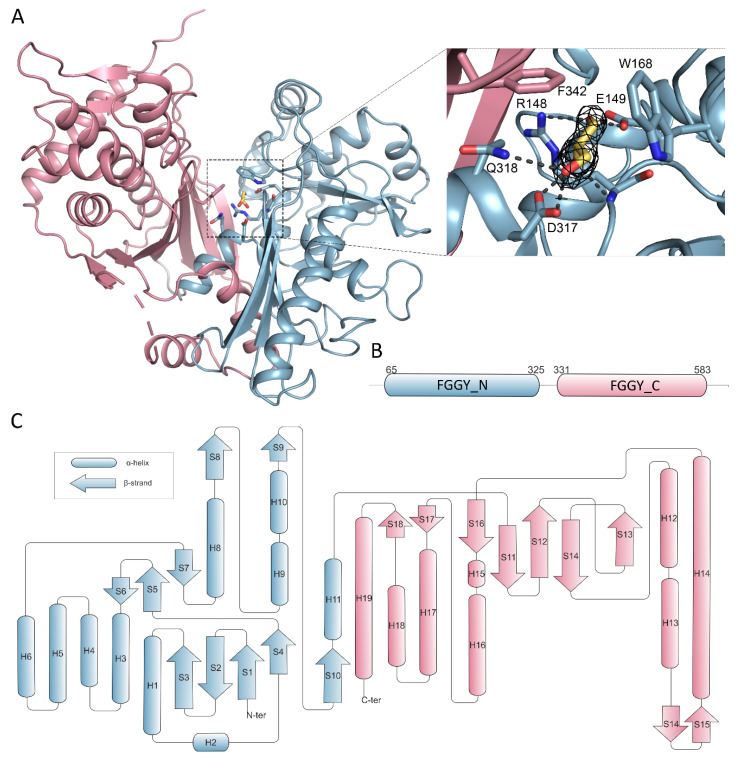
Domain organization of CtGK. Elements constituting N- and C- terminal domains are in blue and pink, respectively. (**A**) Cartoon representation of CtGK monomer; inset shows an enlargement of the glycerol-binding site with 2Fo-fc map contoured at 2σ around the substrate shown as black mesh; ligand and ligand-binding residues shown as sticks. (**B**) Schematic representation of linear domain division. (**C**) Topographic representation of secondary structure elements building the CtGK monomer.

Overall, the CtGK polypeptide chain can be divided into two distinct domains classified by Pfam as FGGY_N (residues 65–325) and FGGY_C (residues 331–583) connected by a flexible region (Figure 5A,B) [25].

**Figure 6:**
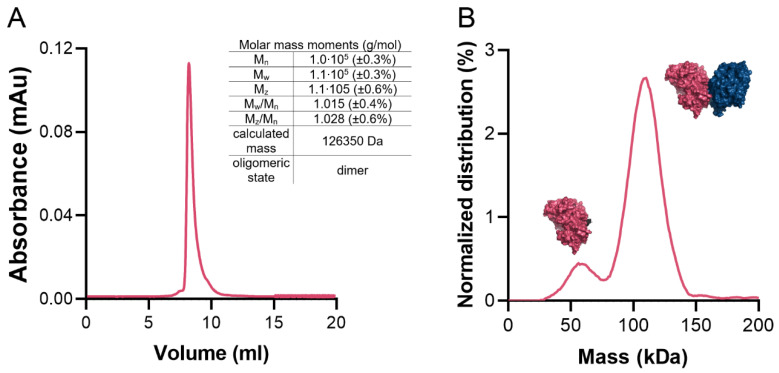
CtGK oligomeric state in solution. (**A**) Size-exclusion chromatography (SEC) elution profile with a table summarizing multi-angle light scattering (MALS) statistics. (**B**) Mass photometry profile. Schematic representations of mono- and dimeric forms of CtGK are inserted next to respective peaks.

The average surface area of the monomer is 20,387 Å^2^ (min. 20,075 Å^2^, max. 21,323 Å^2^). Previous studies have indicated that functional GKs form dimers. A similar case is found in CtGK, which forms extended dimers via the interaction between the C-terminal domains with a prominent contribution from hydrophobic residues (Phe422, Gly436, Phe389, Leu394, Thr437, Phe439, Gly440, Ile441, Thr437, Thr445, Leu438, Ile450, Trp580). On average, the interface area is 1399 Å^2^ (min. 1375 Å^2^, max. 1467 Å^2^), which accounts for only approx. 6.9% of the total protein surface and is predicted by both EPPIC [7] and PDBe PISA [26,27] servers to be biologically significant. To additionally verify the protein’s oligomeric state in the solution, we performed size-exclusion chromatography–multi-angle light scattering (SEC-MALS) and mass photometry analyses (Figure 6A,B) which unequivocally confirmed that CtGK forms dimers in solution as observed in the crystal structure. The small monomeric fraction seen in the mass photometry experiment may be the result of the low protein concentration used and/or the equilibrium conditions.

### 2.3. Glycerol Binding

We initially determined CtGK in an apo form using ethylene glycol as a cryoprotective agent. Subsequently, by replacing the cryoprotectant solution with 20–25% glycerol, we were able to solve the enzyme-substrate complex with a clear electron density that could be attributed to the bound glycerol moiety (Figure 5A). In total, three different crystal structures were obtained in the presence of glycerol, constituting 13 polypeptide chains. Generally, the same extent of the chain could be modeled as in the apo structures, i.e., for residues 65–583 with an approximate 9aa gap between H18 and B18. The substrate is bound in an evolutionarily conserved cleft within the N-terminal domain that opens towards the C-terminal domain (Figure 5A,B). The glycerol molecule is coordinated by polar interactions with side chains of Glu149, Arg 148, Asp317, Gln318, and additionally with Arg148 main-chain amide. The carbon chain of glycerol packs against hydrophobic Trp168 and Phe342 (Figure 5A and Figure S3). A similar mode of binding was described previously for *P. falciparum* GK [16], where Phe271 and Tyr135 undergo shift upon substrate or cofactor binding. However, the movement of equivalent residues (Phe342 and Tyr200) was not observed in either of the presented CtGK complexes.

### 2.4. Nucleotide Binding

**Figure 7:**
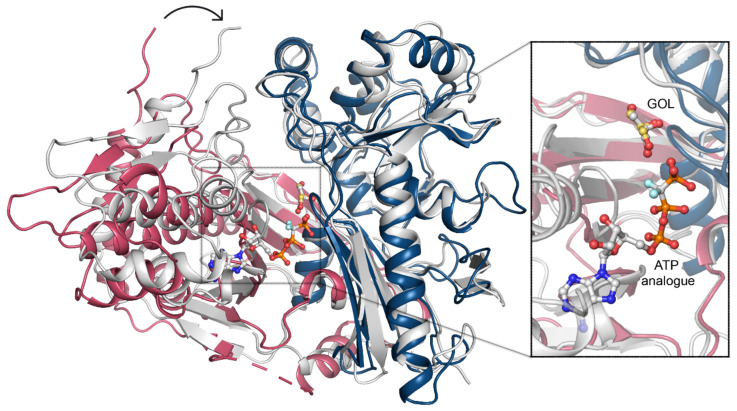
ATP binding. A superposition of CtGK (blue and pink cartoon) and *Escherichia coli* GK (1bwf, white cartoon) with ligands shown in ball-and-stick representation. Inset shows an enlargement of the ligand-binding site.

To uncover the structural determinants of nucleotide-binding by CtGK, we attempted to crystalize either binary CtGK–ATP or ternary CtGK–ATP–glycerol complexes. Despite significant efforts, including co-crystallization or ligand soaking procedures, and testing the known nucleotide-mimicking compounds such as 8-Br-AMP, 5-ITU, or AMPNP [18,28,29], no electron density allowing for reliable nucleotide modeling was identified in any of the analyzed crystals. However, taking into account the high structural similarity between CtGK and PfGK, especially in the active site, we can infer the most likely position of the ATP by comparison of these two structures. In the derived nucleotide-binding model, ATP binds to the gorge between the N- and C-terminal domains with the adenine moiety tightly bound and sugar and phosphate moieties more solvent-exposed (Figure 7).

In this arrangement, the distance between the gamma-phosphate of the nucleotide and glycerol is relatively large (≥4.8 Å) and suggests a necessity for the structure rearrangement for the reaction to occur. Such a closed conformation is likely to occur upon forming a ternary complex (e.g., as presented in Protein Data Bank (PDB) IDs: 1GLL, 1BWF, 1GLJ); however, we could not observe that experimentally [11]. In the ternary complex, the N- and C-terminal domains shift to form a closed structure in which ATP (or its analog) is brought into proximity to glycerol (Figure 7), hence facilitating the phosphate transfer. An additional nucleotide-binding site was described for glycerol kinase from *Trypanosoma brucei gambiense* (PDB ID 3WXL) [30]. Three crucial arginine residues take part in forming this additional ADP-binding site, which however are not conserved in *Chaetomium thermophilum*. Arg22, Arg24, and Arg477 in TbgGK correspond to Glu86, Asn88, and G540 in CtGK, effectively changing the site’s electrostatics and rendering it unlikely to accommodate a negatively charged ligand (Figure S4).

### 2.5. Domain Motion

Previously, two distinct regulatory mechanisms were described for prokaryotic GKs, involving either allosteric regulation or the histidine phosphorylation [13,31]. The common feature of both mechanisms is a structural rearrangement of the two GK domains. The relative domain rearrangement was also suggested to play a pivotal role in the catalytic cycle of GKs in *E. coli* [13,16]. The allosteric regulation was shown not to play a role in so far described eucaryotic GK (16). In *E. coli* GK, phosphorylation of histidine (His232) is one of the regulatory mechanisms, which was not observed in the eukaryotic GKs. In CtGK, the loop corresponding to the *E. coli* “activation loop” bearing the phosphorylatable histidine is hydrophobic and contains no phosphorylatable residues. Our crystallization attempts led to five different structures with a total number of 17 unique CtGK chains. Although crystals were grown from the same condition, the collected datasets were processed in different space groups (see Table 1 for details). Comparison of each of the CtGK subunits indicates a high structural similarity and low Cα root mean square deviation (RMSD) between the domains. The N-terminal, the catalytic domain, is less dynamic than the C-terminal, the dimerization domain (see Table S1). In contrast, the pairwise comparison of entire monomers reveals higher differences of higher magnitude, which can be explained by major conformational rearrangements and changes in the relative orientation of the two protein domains (Video S1, Supplementary Materials). Such a dynamical domain rearrangement, although of a higher amplitude than the one observed herein, seems necessary to bring the ATP in close enough proximity to the GOL to allow the gamma phosphate transfer. In *P. falciparum* GK, the nucleotide is mostly bound by the C-terminal domain, yet one phosphate coordinating residue—Thr268 (Thr339 in CtGK)—is located on the linker between the FGGY_N and FGGY_C domains. In *E. coli*, a flexible arginine was identified to take part in the coordination of one of the phosphates, the corresponding Arg 79 in CtGK belongs to the N-terminal domain, and its side chain is pointed towards the expected ATP molecule. Binding of the nucleotide is likely required for completion of sufficient closure. Consequently, the opening of the monomer would facilitate product release and binding of the fresh substrates.

## 3. Discussion

The product of the glycerol kinase-catalyzed reaction, glycerol 3-phosphate, is employed in the cell either as a precursor for triacylglycerol synthesis or is further oxidized by a flavin adenine dinucleotide (FAD)-dependent glycerol 3-phosphate dehydrogenase to form dihydroxyacetone phosphate. Thus, glycerol kinase links major metabolic pathways such as glycolysis or tricarboxylic acid cycle. Here, we report the first structure of glycerol kinase from the thermophilic filamentous fungus *Chaetomium thermophilum* (CtGK). To date, from all the structures deposited in the PDB, CtGK has the most sequence identity to the human GK. Presented data reveal the mechanism of glycerol binding and shed a light on the substrate-induced domain rearrangements. Of note, we solved and refined five CtGK structures, and each of them belongs to a different space group, despite crystals having grown in the same buffer condition.

Our structural investigations are supplemented with the characterization of CtGK stability, oligomerization, and enzymatic activity. We show that CtGK thermal stability relies on the substrate binding. Our data show that binding of either nucleotide or glycerol alone destabilizes the protein. Only in the presence of two substrates, ATP and glycerol, is CtGK more thermally stable. This may suggest a major structural rearrangement is underway during the binding of only one of the substrates and that the protein in the process of catalysis, when both substrates are bound, adopts the most stable conformation. We observed that in the absence of nucleotide, CtGK adopts open conformation, which upon nucleotide binding closes up. We hypothesize that, when only one substrate (GOL, ADP, or ATP) is present, the conformation is not stable and can only be stabilized and closed in the presence of both, i.e., nucleotide and GOL. Similar conformational rearrangements were observed and verified in the solution e.g., for ADP-dependent glucokinase [32]. By analogy, it is likely that also in CtGK the structural dynamics is larger than could be observed in static crystal structures. However, confirmation of rearrangements’ magnitude would require follow-up solution studies such as SAXS and likely contribute little to the overall understanding of the catalytic mechanism of kinases.

Previous studies have shown that FBP can interact with *E. coli* GK, impairing its activity and leading to protein tetramerization. In contrast, *Trypanosoma brucei gambiense* GK is insensitive to FBP regulation [30]. Here, we demonstrate that FBP neither inhibits CtGK nor has an influence on its oligomerization state. To further investigate the FBP binding by CtGK, we compared its structure with the *E. coli* homolog. In EcGK (PDB ID 1BO5), the side chain of Arg236 and the main chain of Gly234, both located on the surface loop and protruding towards a neighboring monomer, are essential for the binding of FBP. These correspond to Gly308 and Gly304 in CtGK and the respective loop is tightly associated with the core of the protein (Figure S5). This renders FBP binding in a manner similar to the one observed in EcGK highly unlikely. However, as the results of our thermal shift assay show the ability of FBP to stabilize CtGK, we cannot exclude that some yet unidentified binding site is present in CtGK. Moreover, as we observed the substrate inhibition by ATP, we cannot rule out the existence of the second nucleotide-binding site; however, its location may be different from the one described in TbgGK. 

Motivated by our previous works, we also demonstrated that ADPGK and ADPGK/PFK from hyperthermophilic archaea can be used to measure the ATPase activity of glycerol kinase. The developed assay is quick and robust, and it can successfully replace the method based on the pyruvate kinase/lactate dehydrogenase-coupled assay. However, we observed subtle differences between calculated kinetic values for different assays that may reflect the yet unknown interplay between used enzymes, substrates, and/or buffer composition. Thus, the use of a direct measurement of ATP consumption, employing, e.g., a radiolabeled nucleotide analog, could serve as a validation tool for both coupled assays used in our study. However, both types of coupled enzymatic assays are simple and robust, hence they are ideal for rapid quantification of GK activity. Additionally, as the ADPGK-coupled assay was already successfully used for the measurement of creatine kinase activity, we hypothesize that it can be employed to assess other ATP-dependent enzymes such as protein kinases. Nevertheless, it is the scope of future developments not related to the research presented in our work. 

In summary, our study provided structural insights into the glycerol phosphorylation in the thermophilic eukaryote *Chaetomium thermophilum*. Knowledge of the CtGK molecular architecture and mechanism of glycerol phosphorylation will allow to further understand glycerol metabolism in fungi and eukaryotes in general.

## 4. Materials and Methods

### 4.1. Protein Expression and Purification

The *Chaetomium thermophilum* gene encoding glycerol kinase (residues 67–590) was codon-optimized for improved expression in *E. coli*. The gene with an N-terminal His-Tag followed by the tobacco etch virus (TEV) recognition site was synthesized (GenScript, Leiden, Netherlands) and subcloned to a pET24d expression vector. N-terminal His-tagged CtGK was expressed in *E. coli* BL21 (DE3) cultured in terrific broth medium supplemented with kanamycin. Briefly, cells were cultivated at 37 °C until OD_600_ value of 1 and induced with 0.5 mM isopropyl-β-d-thiogalactoside (IPTG). The cultures were incubated for 16 h at 16 °C. Subsequently, cells were collected and resuspended in the lysis buffer containing 20 mM HEPES, pH 8.0, 150 mM NaCl, and 20 mM imidazole, and lysed by sonication. The soluble fraction was loaded onto a Ni-Sepharose (GE Healthcare, Uppsala, Sweden) column equilibrated with lysis buffer. Protein was eluted with a similar buffer with 500 mM imidazole concentration and mixed with TEV protease. After overnight incubation, the solution was loaded onto Ni-Sepharose resin to remove the His-tag and TEV protease. Finally, CtGK was concentrated and purified to homogeneity by gel filtration using Superdex 75 (GE Healthcare, Uppsala, Sweden) in 20 mM HEPES, pH 8.0, and 150 mM NaCl. Fractions containing protein of interest were pooled and concentrated to 10 mg/mL.

MjADPGK and phADPGK/PFK were obtained as previously described [18,28]. Briefly, genes encoding full-length mjADPGK and phADPGK were synthesized (GenScript, Leiden, Netherlands) and cloned into pET24d and pET15b plasmid, respectively. The expression was performed in *E. coli* and the proteins were purified to homogeneity in the buffer (20 mm HEPES, pH 8.0, 150 mm NaCl, and 5 mm MgCl_2_).

### 4.2. Thermal Stability Assay

Melting points (Tm values) of CtGK were obtained using a thermal shift assay (TSA) as described previously [17]. Protein (2.5 mg/mL) was incubated with Sypro Orange dye in 50 mM TRIS and 200 mM NaCl, pH 8.0. For determination of the GOL/ATP/ADP/FBP impact on the protein stability, the reaction buffer was supplemented with 5% GOL and/or 0.5 mM ATP/ADP/FBP. The fluorescence signal of Sypro Orange was determined as a function of temperature between 5 and 95 °C in increments of 1.2 °C/min. The melting temperature was calculated as the inflection point of the fluorescence vs. temperature function. Each experiment was carried out at least in triplicate.

### 4.3. CtGK Activity Assays

#### 4.3.1. Pyruvate Kinase/Lactate Dehydrogenase (PK/LDH)-Coupled Activity Assay

CtGK activity was evaluated by monitoring the level of one of the reaction products (pyruvate) in a pyruvate kinase/lactate dehydrogenase-coupled system [33]. The reaction is initiated by adding different concentrations of ATP. NADH oxidation by lactate dehydrogenase is monitored spectrophotometrically at 340 nm. Activity assays were performed in 96 well-format spectrophotometric plates using a TECAN microplate UV–VIS reader (Infinite 200 Pro, Tecan, Männedorf, Switzerland), at 37 °C in reaction buffer (20 mM Tris-HCl, pH 7.4, 250 mM sucrose, 50 mM, KCl, and 5 mM MgCl_2_) supplemented with CtGK (3 µg), pyruvate kinase (10 µg), lactate dehydrogenase (10 µg), glycerol (1 mM), PEP (1 mM), and NADH (0.5 mM). The reaction was initiated by adding different concentrations of ATP.

#### 4.3.2. ADP-Dependent Glucokinase/Glucose-6-Phosphate Dehydrogenase (ADPGK/G6PD)-Coupled Activity Assay

CtGK activity was assessed by monitoring the level of one of the reaction products (glucose-6-phosphate) in a coupled ADP-specific glucokinase/glucose-6-phosphate dehydrogenase (G6PDH) reaction essentially as described previously [9,18,28]. The reaction was initiated by the addition of different concentrations of ATP. NADP reduction by G6PDH was monitored spectrophotometrically at 340 nm as mentioned above. The reaction buffer was supplemented with either mjADPGK or phADPGK (1 µg), glucose (1 mM), glucose-6-phosphate dehydrogenase (1U), and NADP (0.5 mM).

### 4.4. Determination of Oligomeric State in Solution

The oligomeric states of CtGK were investigated by size-exclusion chromatography coupled to multi-angle light scattering (MALS) as described previously [34]. Briefly, the pure protein sample was separated on an XBridge BEH200 7.8 × 300 column (Waters, Milford, MA, USA) in 50 mM Tris, pH = 7.8, and 200 mM NaCl buffer at a flow rate of 1 mL min^−1^. Results were analyzed using ASTRA software (version 6, Wyatt Technology, Santa Barbara, CA, USA). Additionally, the protein sample was analyzed by mass photometry [35]. Mass photometry data were collected on a Refeyn OneMP instrument (Refeyn, Oxford, UK). The instrument was calibrated with a mix of BSA and urease. For each measurement, a 10 μL amount of CtGK was applied to 10 μL SEC buffer on a coverslip, resulting in a final concentration of 1–10 nM. Movies were acquired using AcquireMP v2.2.1. software (Refeyn) for 120 s with a frame rate of 1000 Hz with a further frame binning of 10. All data were processed in DiscoverMP v2.2.1 software (Refeyn). Masses were estimated by fitting a Gaussian distribution into mass histograms.

### 4.5. Crystallization, Data Collection, and Structure Determination

The initial screening for crystallization conditions was performed using a variety of available commercial screens at the Structural Biology Core Facility (Kraków, Poland, www.structuralbiology.pl). The first crystals of CtGK were obtained by the sitting-drop vapor diffusion technique at 293 K using a commercially available buffer set (SG1, Molecular Dimensions Sheffield, UK) from a condition B5 containing 0.2 M CaCl_2_ and 20% PEG 3350. After crystallization condition optimization, diffraction quality crystals were obtained by mixing 10 mg/mL protein in a 1:1 ratio with 0.2 M CaCl_2_ and 18% PEG 3350. Crystals were transferred to the solution containing 25% of glycerol or ethylene glycol soaked for 1–5 min and flash cooled in liquid nitrogen.

Diffraction data were collected using the MX-beamline 14.1 at the BESSY II (HZB, Berlin, Germany) [36], PXI at SLS (Villigen, Switzerland) [37], and P11 at DESY (Hamburg, Germany) [38] electron storage rings. The diffraction data were processed using XDS as implemented in the XDSAPP package [39]. The structure was initially solved using the MoRDa server [24] and Phaser [40] using PDB ID: 5AZI [15] as a search model. The obtained models were manually rebuilt using *Coot* [41] and refined using phenix.refine [42]. Water molecules were automatically placed during structure refinement, further added using Coot, and finally manually inspected. The quality of the model was validated using MolProbity [43]. All structures were determined to high resolution and refined to low values of R_work_ and R_free_, which are indicators of good accuracy of the models. The data collection, structure refinements, and validation statistics are summarized in Table 1. The analysis and comparison of structures were performed in PyMOL (Molecular Graphics System, Version 2.0 Schrödinger, LLC, New York, NY, USA).

## Figures and Tables

**Table 1:** Data collection and refinement statistics. Statistics for the highest-resolution shell are shown in parentheses.

PDB ID	6ZQ4	6ZQ5	6ZQ6	6ZQ7	6ZQ8
Light Source	BESSY 14.1	BESSY 14.1	BESSY 14.1	SLS PXI	DESY P11
Wavelength (Å)	0.918	0.918	0.918	1.00	1.033
Resolution range (Å)	23.01–2.02 (2.07–2.02)	46.71–2.14 (2.27–2.14)	48.6–2.30 (2.44–2.30)	44.72–2.42 (2.51–2.42)	47.04–2.38 (2.53–2.38)
Space group	P_1_	P222_1_	P2_1_2_1_2	I222	P3_2_21
Unit cell (Å, °)	61.38 112.82 179.54 85.77 90.04 85.45	63.57 111.47 171.25 90.0 90.0 90.0	170.73 222.03 61.33 90.0 90.0 90.0	88.60 89.44 131.29 90.0 90.0 90.0	92.14 92.14 232.95 90.0 90.0 120.0
Total reflections	1906807 (71895)	374918 (38178)	383976 (62534)	40292 (3843)	888970 (135787)
Unique reflections	307534 (19548)	61278 (8088)	103148 (16723)	20156 (1926)	46200 (6995)
Multiplicity	6.2 (3.68)	6.12 (4.72)	3.72 (3.74)	2.0 (2.0)	19.24 (19.41)
Completeness (%)	97.4 (83.6)	89.9 (74.4)	98.5 (98.1)	99.25 (96.28)	98.8 (94.3)
Mean I/sigma(I)	4.85 (0.74)	11.59 (0.67)	6.80 (0.77)	17.17 (1.44)	17.95 (1.49)
Wilson B-factor	36.82	47.97	47.10	75.60	66.89
R-merge (%)	21.9 (147.4)	11.9 (219.7)	17.7 (159.1)	1.76 (36.24)	12.4 (196.4)
R-meas (%)	23.7 (171.7)	13.0 (243.5)	20.7 (185.0)	2.48 (51.25)	12.7 (201.7)
CC1/2 (%)	99.1 (26.9)	99.8 (27.6)	99.3 (33.1)	100 (80.6)	99.9 (63.3)
Reflections used in refinement	306624 (28330)	61158 (4981)	103082 (10153)	20131 (1931)	46193 (4159)
Reflections used for R-free	3221 (298)	2096 (171)	2099 (207)	1008 (96)	2099 (189)
R-work (%)	20.49	21.55	21.47	18.64	24.36
R-free (%)	23.87	24.95	24.91	25.13	28.66
Number of protein chains in ASU	8	2	4	1	2
Number of non-hydrogen atoms	33032	8167	16060	3995	7887
macromolecules	31248	7801	15603	3912	7809
ligands	78	44	46	10	4
solvent	1706	322	411	73	74
Protein residues	4085	1021	2041	510	1021
RMS (bonds)	0.009	0.004	0.006	0.008	0.004
RMS (angles)	1.05	0.68	0.87	1.03	0.66
Ramachandran favored (%)	97.16	97.24	96.64	95.45	96.05
Ramachandran allowed (%)	2.76	2.76	3.06	4.36	3.95
Ramachandran outliers (%)	0.07	0	0.30	0.2	0
Rotamer outliers (%)	0.40	1.34	0.85	0.49	0.49
Clashscore	6.42	3.71	3.95	12.65	9.07
Average B-factor	51.66	53.36	51.92	93.07	79.91
macromolecules	51.84	53.42	51.98	93.29	80.12
ligands	42.64	62.71	52.98	75.4	53.78
solvent	48.90	50.69	49.33	83.77	59.43

## Supplementary Materials

Supplementary Materials can be found at https://www.mdpi.com/1422-0067/21/24/9570/s1.

## Abbreviations

5-ITU	5-Iodotubercidin
8-Br-AMP	8-Bromoadenosine 5′-monophosphate
AMPNP	Adenosine-5′-[(α,β)-imido]diphosphate
ADPGK	ADP-dependent glucokinase
ADPGK/PFK	ADP-dependent gluco/phosphofructokinase
ASU	Asymmetric unit
CtGK	*Chaetomium thermophilum* glycerol kinase
FAD	Flavin adenine dinucleotide
FBP	Fructose-1,6-bisphoshpate
G3P	Glycerol 3-phosphate
G6PD	Glucose-6-phosphate dehydrogenase
GOL	Glycerol
GK	Glycerol kinase
LDH	Lactate dehydrogenase
MALS	Multi-angle light scattering
NAD/NADH	Nicotinamide adenine dinucleotide
NADP/NADPH	Nicotinamide adenine dinucleotide phosphate
PDB	Protein Data Bank
PfGK	*Plasmodium falciparum* glycerol kinase
PK	Pyruvate kinase
SEC	Size-exclusion chromatography
