# Supplementary material for: Structural Characterization of Glycerol Kinase from the Thermophilic Fungus Chaetomium thermophilum
Source: Int J Mol Sci. 2020 Dec 16;21(24):9570. doi: 10.3390/ijms21249570 (PMC7765489; doi:10.3390/ijms21249570)
Supplement: Supplementary file 1 [file ijms-21-09570-s001.zip › Supplement_FINAL.pdf]

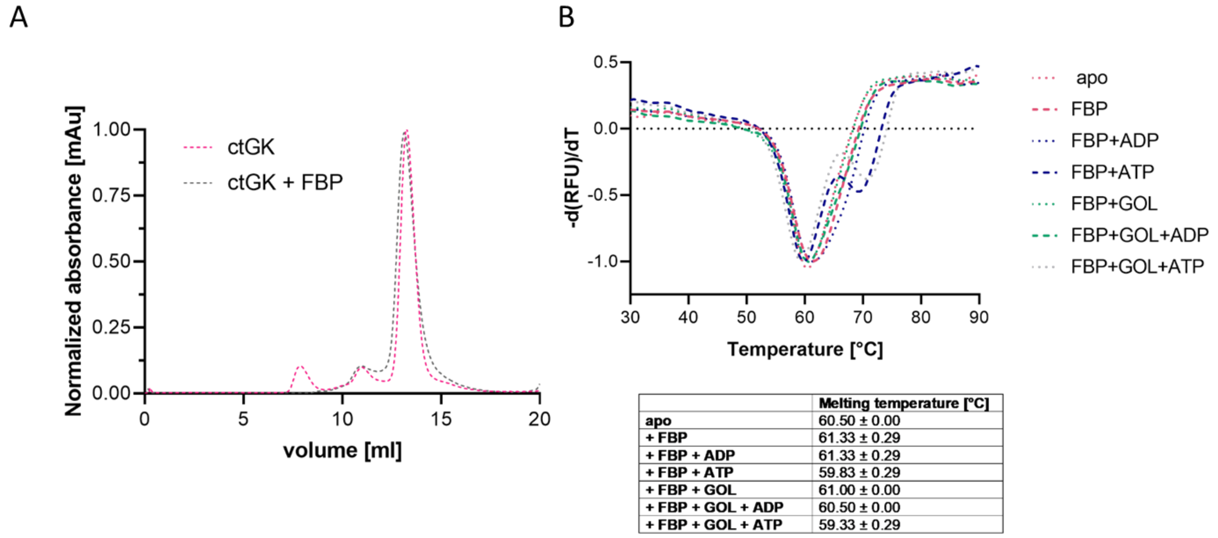

	Melting temperature [°C]
apo	60.50 ± 0.00
+ FBP	61.33 ± 0.29
+ FBP + ADP	61.33 ± 0.29
+ FBP + ATP	59.83 ± 0.29
+ FBP + GOL	61.00 ± 0.00
+ FBP + GOL + ADP	60.50 ± 0.00
+ FBP + GOL + ATP	59.33 ± 0.29

Figure S1. Influence of fructose 1,6-bis-phosphate (FBP) on ctGK oligomerization and stability. A. Size-exclusion profiles of ctGK with and without FBP. B. Thermal shift assay of ctGK in the presence of FBP and different substrates.

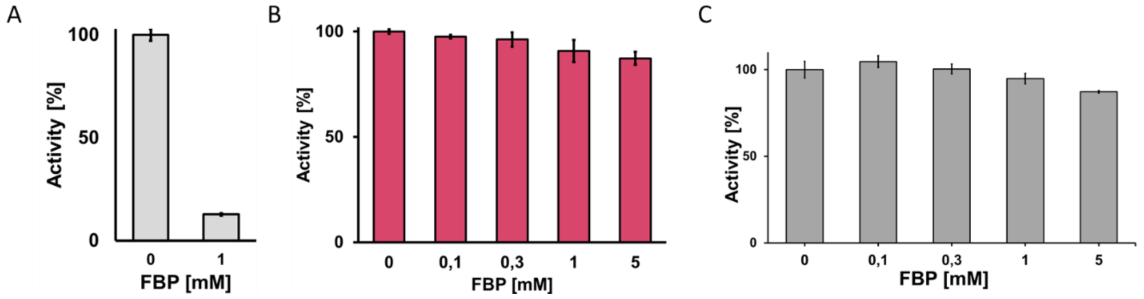

Figure S2. Fructose 1,6-bis-phosphate does not inhibit ctGK. A. Inhibition of mjADPGK/PFK by FBP. B. Inhibition of ctGK activity in the presence of FBP. C. phADPGK activity in the presence of FBP.

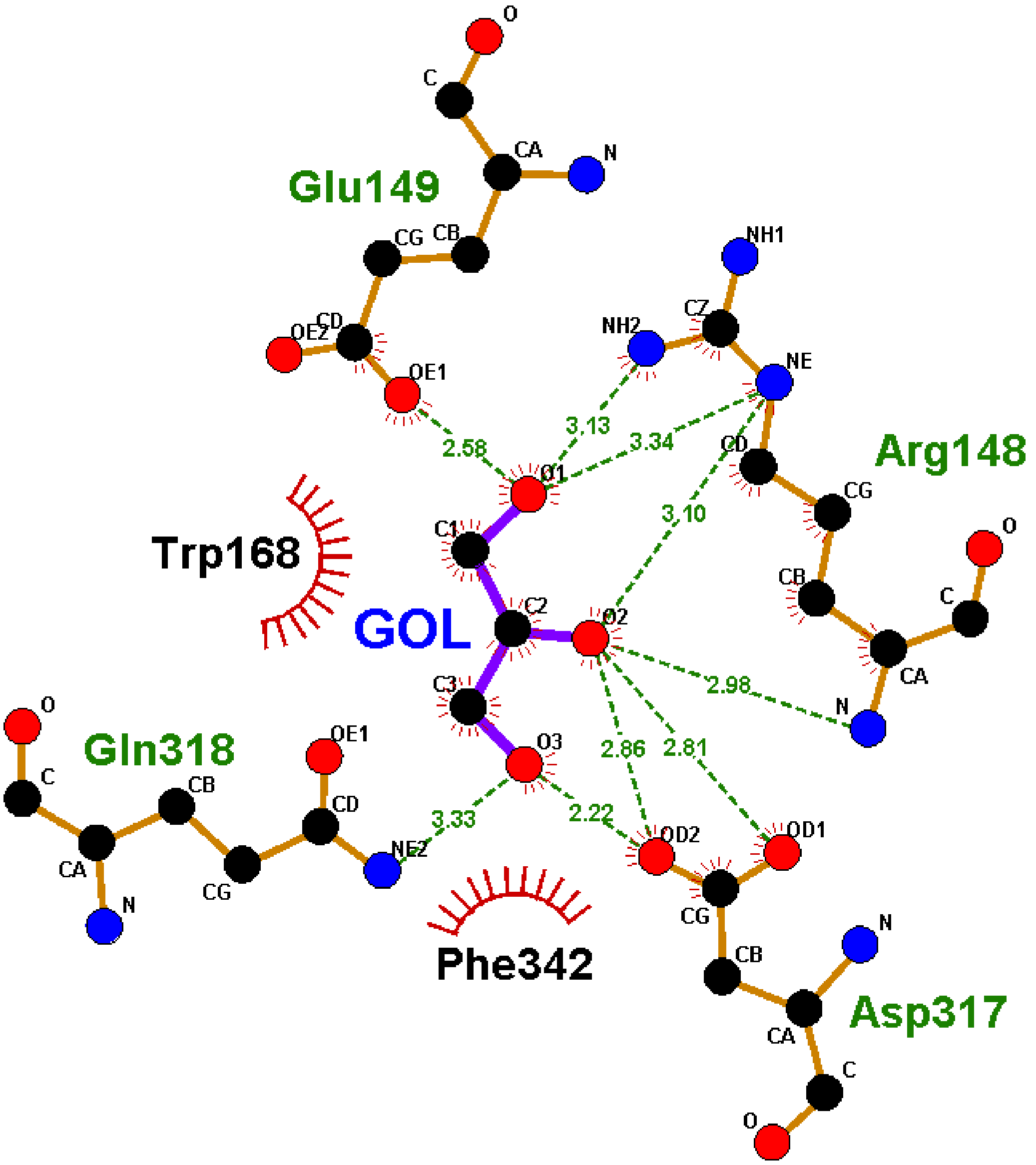

Figure S3. Ligplot showing the protein-glycerol interactions, based on energy score (hydrogen bonding and hydrophobic), generated by Ligplot program.

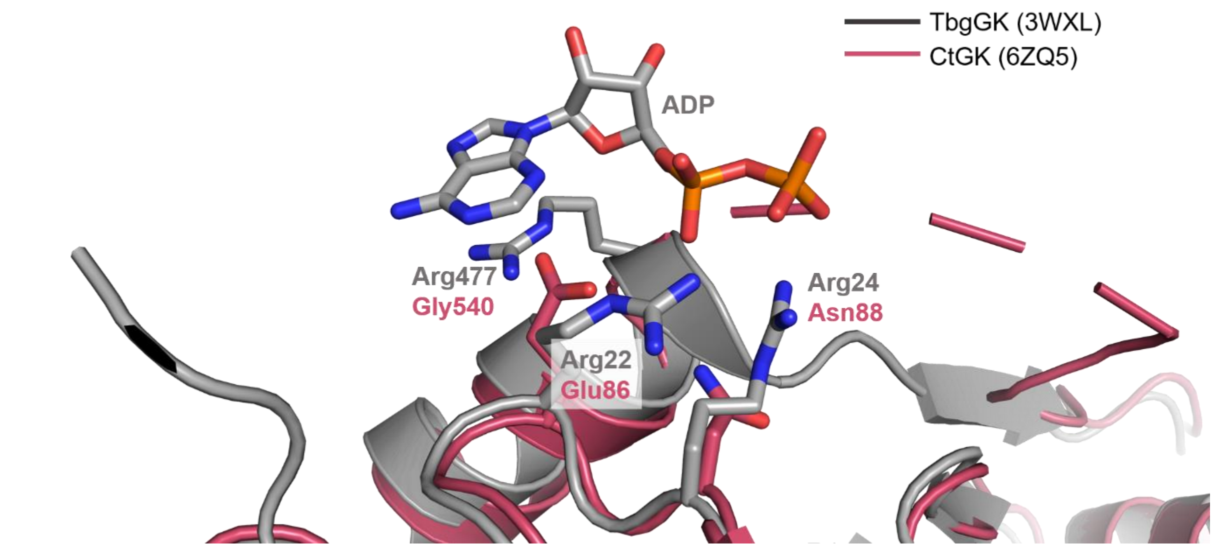

Figure S4. Superposition of CtGK (dark pink) and TbgGK (grey) secondary nucleotide-binding site.

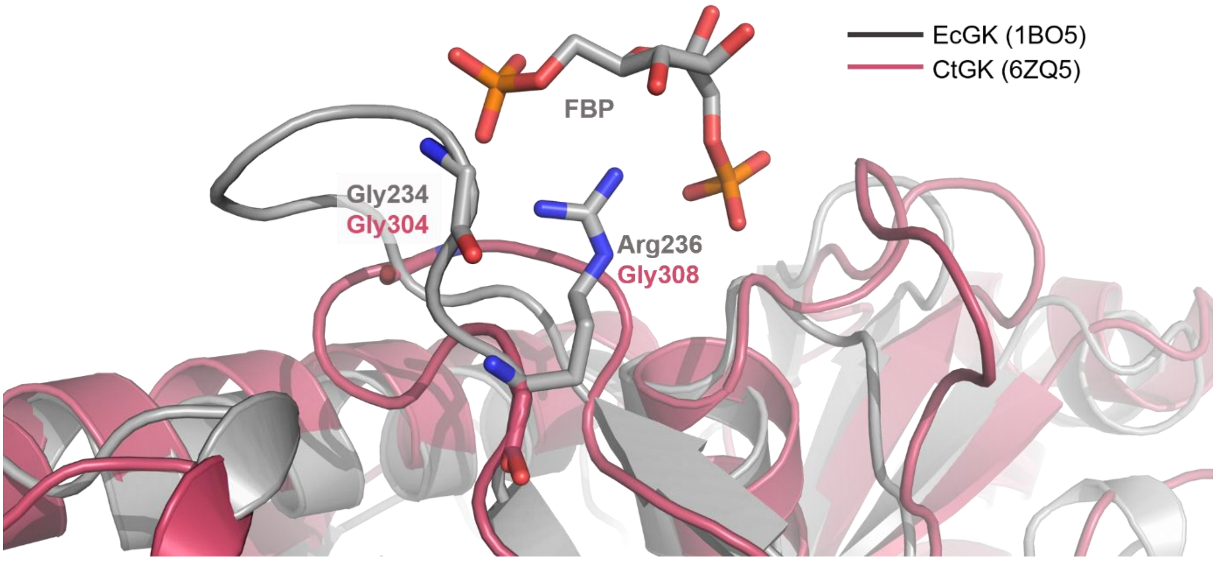

Figure S5. Superposition of ctGK (dark pink) and EcGK (grey) FBP binding site.

Table S1. Mutual differences between CtGK monomers expressed as main chain RMSD [Å] for (A) full chain, (B) N-terminal domain, (C) C-terminal domain. Values obtained using Cealign in PyMol. In bold are highlighted complexes containing GOL in the active site.

A

Model	Chain	**6ZQ4**								6ZQ5		**6ZQ6**				**6ZQ7**	6ZQ8	
		A	B	C	D	E	F	G	H	A	B	A	B	C	D	A	A	B
**6ZQ4**	A		0,736	0,313	0,279	0,302	0,312	0,217	0,276	0,375	0,442	0,371	0,688	0,331	0,336	1,030	1,762	0,570
	B			0,811	0,321	0,331	0,287	0,366	0,365	0,816	0,861	0,860	0,488	0,373	0,337	0,845	1,721	0,543
	C				0,791	0,271	0,328	0,228	0,472	0,367	0,560	0,382	0,629	0,824	0,825	1,193	2,042	0,554
	D					0,177	0,219	0,251	0,389	0,360	0,513	0,449	0,388	0,396	0,395	1,039	1,937	0,572
	E						0,246	0,268	0,412	0,352	0,528	0,469	0,429	0,411	0,418	1,001	1,920	0,595
	F							0,286	0,361	0,390	0,480	0,454	0,431	0,371	0,363	0,935	1,843	0,590
	G								0,345	0,342	0,477	0,353	0,373	0,317	0,346	0,903	1,848	0,581
	H									0,467	0,352	0,476	0,560	0,440	0,388	0,720	1,553	0,574
6ZQ5	A										0,447	0,507	0,709	0,469	0,497	1,177	1,918	0,653
	B											0,545	0,870	0,554	0,531	1,010	1,507	0,632
**6ZQ6**	A												0,691	0,243	0,275	1,025	1,747	0,488
	B													0,332	0,355	0,987	1,881	0,556
	C														0,182	0,812	1,713	0,452
	D															0,757	1,675	0,467
**6ZQ7**	A																1,183	0,813
6ZQ8	A																	1,533
	B																	

Av	1/2 SD	min	max
0,657	0,228	0,177	2,042

B

Model	Chain	**6ZQ4**								6ZQ5		**6ZQ6**				**6ZQ7**	6ZQ8	
		A	B	C	D	E	F	G	H	A	B	A	B	C	D	A	A	B
**6ZQ4**	A		0,189	0,193	0,174	0,218	0,218	0,186	0,230	0,319	0,371	0,228	0,214	0,199	0,217	0,423	0,714	0,394
	B			0,204	0,175	0,216	0,193	0,213	0,241	0,329	0,374	0,244	0,226	0,222	0,221	0,436	0,714	0,389
	C				0,176	0,204	0,201	0,175	0,241	0,304	0,358	0,212	0,216	0,203	0,229	0,403	0,692	0,357
	D					0,192	0,181	0,196	0,219	0,329	0,361	0,229	0,217	0,219	0,219	0,419	0,712	0,380
	E						0,192	0,220	0,243	0,338	0,382	0,246	0,228	0,233	0,240	0,432	0,687	0,385
	F							0,216	0,228	0,322	0,387	0,240	0,228	0,223	0,221	0,447	0,717	0,408
	G								0,231	0,300	0,351	0,208	0,219	0,207	0,231	0,412	0,706	0,361
	H									0,301	0,340	0,255	0,250	0,258	0,260	0,408	0,694	0,368
6ZQ5	A										0,284	0,254	0,307	0,292	0,311	0,416	0,704	0,372
	B											0,318	0,363	0,378	0,380	0,468	0,674	0,377
**6ZQ6**	A												0,211	0,182	0,210	0,401	0,707	0,345
	B													0,185	0,186	0,427	0,691	0,394
	C														0,149	0,421	0,701	0,388
	D															0,447	0,683	0,405
**6ZQ7**	A																0,766	0,431
6ZQ8	A																	0,692
	B																	

Av	1/2 SD	min	max
0,334	0,079	0,149	0,766

C

Model	Chain	**6ZQ4**								6ZQ5		**6ZQ6**				**6ZQ7**	6ZQ8	
		A	B	C	D	E	F	G	H	A	B	A	B	C	D	A	A	B
**6ZQ4**	A		0,257	0,258	0,222	0,191	0,229	0,173	0,192	0,203	0,317	0,351	0,357	0,300	0,304	0,530	0,998	0,460
	B			0,350	0,296	0,251	0,156	0,258	0,241	0,315	0,347	0,366	0,378	0,255	0,240	0,460	0,879	0,341
	C				0,179	0,207	0,276	0,184	0,376	0,275	0,469	0,287	0,193	0,294	0,307	0,684	1,123	0,443
	D					0,120	0,207	0,183	0,317	0,244	0,393	0,318	0,261	0,291	0,299	0,625	1,059	0,448
	E						0,176	0,167	0,275	0,214	0,346	0,328	0,287	0,283	0,287	0,588	1,013	0,420
	F							0,201	0,252	0,217	0,330	0,326	0,311	0,232	0,230	0,514	0,916	0,344
	G								0,251	0,209	0,354	0,298	0,274	0,267	0,281	0,578	1,009	0,406
	H									0,273	0,269	0,388	0,442	0,332	0,337	0,448	0,883	0,462
6ZQ5	A										0,283	0,366	0,352	0,340	0,351	0,586	0,989	0,460
	B											0,477	0,520	0,427	0,436	0,482	0,831	0,503
**6ZQ6**	A												0,245	0,257	0,268	0,605	1,034	0,418
	B													0,276	0,280	0,693	1,112	0,407
	C														0,150	0,484	0,943	0,314
	D															0,496	0,952	0,347
**6ZQ7**	A																0,714	0,555
6ZQ8	A																	0,878
	B																	

Av	1/2 SD	min	max
0,407	0,117	0,120	1,123

RMSD [Å]

0 — 2
